# A Bayesian multilevel model for populations of networks using exponential-family random graphs

**DOI:** 10.1007/s11222-024-10446-0

**Published:** 2024-06-19

**Authors:** Brieuc Lehmann, Simon White

**Affiliations:** 1 Department of Statistical Science, University College London, 1-19 Torrington Place, London, WC1e 7HB UK; 2 Department of Psychiatry, University of Cambridge, Cambridge, CB2 0AH UK; 3 MRC Biostatistics Unit, University of Cambridge, Cambridge, CB2 0SR UK

**Keywords:** Exponential random graph model (ERGM), Bayesian linear regression, Markov chain Monte Carlo (MCMC), Brain networks

## Abstract

The collection of data on populations of networks is becoming increasingly common, where each data point can be seen as a realisation of a network-valued random variable. Moreover, each data point may be accompanied by some additional covariate information and one may be interested in assessing the effect of these covariates on network structure within the population. A canonical example is that of brain networks: a typical neuroimaging study collects one or more brain scans across multiple individuals, each of which can be modelled as a network with nodes corresponding to distinct brain regions and edges corresponding to structural or functional connections between these regions. Most statistical network models, however, were originally proposed to describe a single underlying relational structure, although recent years have seen a drive to extend these models to populations of networks. Here, we describe a model for when the outcome of interest is a network-valued random variable whose distribution is given by an exponential random graph model. To perform inference, we implement an exchange-within-Gibbs MCMC algorithm that generates samples from the doubly-intractable posterior. To illustrate this approach, we use it to assess population-level variations in networks derived from fMRI scans, enabling the inference of age- and intelligence-related differences in the topological structure of the brain’s functional connectivity.

**Supplementary Information:**

The online version contains supplementary material available at 10.1007/s11222-024-10446-0.

## Introduction

The statistical analysis of network data is becoming increasingly commonplace, with applications across various disciplines, such as epidemiology, social science, neuroscience and finance (Kolaczyk 2009). Over the last four decades, a number of statistical models for networks have been developed, including stochastic blockmodels (Holland et al. 1983), latent space models (Hoff et al. 2002) and—the focus of this article—exponential random graph models (ERGMs) (Frank and Strauss 1986).

An exponential random graph model is a set of parametric statistical distributions on network data (see Schweinberger et al. (2020) for a recent review). The aim of the model is to characterise the distribution of a network in terms of a set of *summary statistics*. These summary statistics are typically comprised of topological features of the network, such as the number of edges and subgraph counts. The summary statistics enter the likelihood via a weighted sum; the weights are (unknown) model parameters that quantify the relative influence of the corresponding summary statistic on the overall network structure and must be inferred from the data. ERGMs are thus a flexible way in which to describe the global network structure as a function of network summary statistics.

To date, statistical network models, including ERGMs, have largely focused on the analysis of a single network. Formally, a network consists of a set of nodes and a set of edges between these nodes. Let $\mathcal {N} = \lbrace 1, \dots , N \rbrace$ be a finite set of nodes, each of which may be associated with covariates $x_i \in \mathcal {X} \subseteq \mathbb {R}^q$. An edge from node *i* to node *j* is denoted by $Y_{ij}$, so that the network is encoded by the adjacency matrix $\pmb {Y} = (Y_{ij})_{i,j \in \mathcal {N}}$. For our purposes, the set of nodes $\mathcal {N}$ and their covariates $\pmb {x} = \lbrace x_1, \dots , x_N \rbrace$ are considered fixed, while the edges are considered to be random variables. Denote $\pmb {y}$ to be an instantiation, or outcome, of the random adjacency matrix $\pmb {Y}$ and write $\mathbb {P}(\pmb {Y} = \pmb {y}):= \pi (\pmb {y})$ for the probability that $\pmb {Y}$ takes the value $\pmb {y}$. A statistical network model specifies a parametrised probability distribution on the adjacency matrix $\pi (\pmb {y} | \pmb {x}, \theta )$ where $\theta$ is a vector of model parameters.

A population of networks consists of $n > 1$ adjacency matrices $\pmb {Y}^{(1)}, \dots , \pmb {Y}^{(n)}$ defined on a common set of nodes $\mathcal {N}$. We will assume for simplicity that the nodal covariates are the same across networks, though in principle this not need be the case. A common example of a population of networks arises in neuroimaging, where a typical study consists of brain data across a number of participants, each constituting an individual network. Network analyses of the brain can provide insight into cognitive function by revealing how distinct brain areas work in conjunction (Fuster 2006). These analyses aim to identify salient topological features of the brain’s connectivity structure that are common across individuals or that vary with a given covariate.

While one could fit a single model to each individual network separately, it is not straightforward to combine these individual results into a single coherent result that is representative of the whole population. An alternative approach is to construct a group-representative network by, for example, taking the mean of the edges across the individual networks and applying a threshold to the resulting weighted network (Achard et al. 2006). These approaches ignore the individual variability present in the networks and, moreover, typically do not accurately summarise the topological information across the individual networks (Ginestet et al. 2011).

A more statistical approach is to treat each individual networks as distinct statistical units arising from a joint probability distribution $\pi (\pmb {y}^{(1)}, \dots , \pmb {y}^{(n)}| \pmb {x}, \theta )$ (Ginestet et al. 2017). Here, we describe how to perform Bayesian linear regression where the outcome of interest is a network-valued random variable whose distribution is described by an exponential random graph models. By modelling the networks jointly, this framework provides a principled approach to characterise the relational structure for an entire population, and allows one to assess how network structure varies with a given set of network-level covariates. In the case of binary covariates, our method can be used to infer group-level differences in the network structure between *sets of networks*. We demonstrate on both simulated networks and real networks derived from resting-state functional magnetic resonance imaging (fMRI) scans from an ageing study.

Inference for Bayesian ERGMs is challenging due to the double-intractability of the ERGM posterior distribution; standard Markov chain Monte Carlo (MCMC) schemes such as the Metropolis algorithm are not feasible as it is not possible to evaluate the acceptance ratio. A common workaround is to apply the exchange algorithm (Murray et al. 2006), which was first employed in the context of Bayesian ERGMs by Caimo and Friel (2011). To perform inference for our framework for populations of networks, we implemented an *exchange-within-Gibbs* algorithm that combines the exchange algorithm with the Gibbs sampler (Geman and Geman 1984) to produce samples from the target posterior distribution. The parameterization of general multilevel models can play an important role in the overall efficiency of a MCMC scheme (Gelfand et al. 1995; Papaspiliopoulos et al. 2003, 2007). To improve the mixing properties of the algorithm, we use an ancillarity-sufficiency interweaving strategy (ASIS) that interweaves between the *centered* and non-centered parameterizations (Yu and Meng 2011). To further boost efficiency, we also employ adaptation of the random-walk proposal parameters in the algorithm (see e.g. Roberts et al. (1997)).

### Related work

Our work builds on that of Slaughter and Koehly (2016) who studied multiple approaches to building Bayesian hierarchical models for populations of networks based on ERGMs, including an example of Bayesian linear regression with a single covariate per network. We extend the approach of Slaughter and Koehly (2016) to explicitly handle multiple network-level covariates, employing a matrix Normal prior on the regression coefficients which admits (partial) conjugacy. As noted by Slaughter and Koehly (2016), multilevel models frequently exhibit poor mixing for some of the parameters. Our use of the ASIS algorithm greatly improves the efficiency of the sampler, allowing us to perform linear regression on larger populations of networks, and with a larger number of nodes in each network. We now describe some alternative approaches to modelling populations of networks.

### Hierarchical ERGMs

**Fig. 1:**
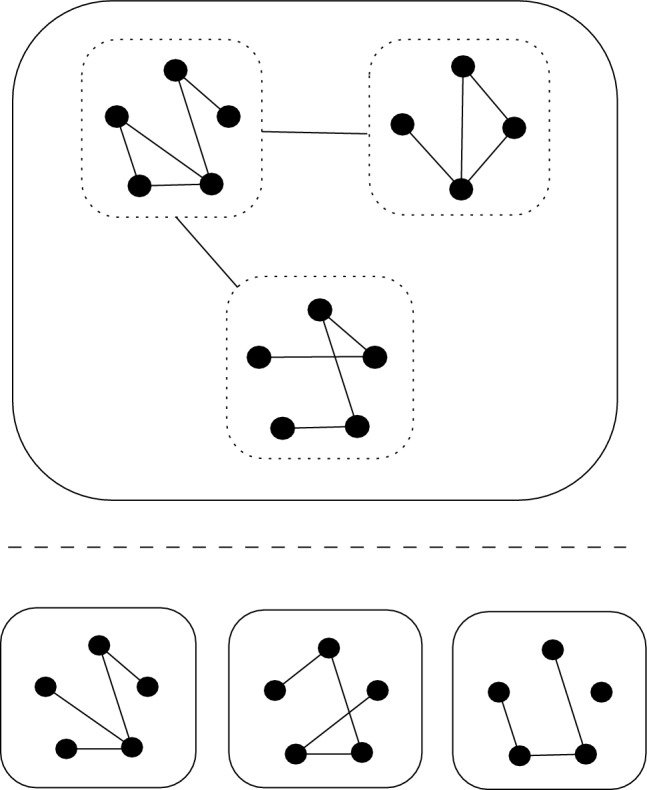
A population of networks (bottom) can be seen as a special case of a multilevel network (top) in which each subset of nodes contains the same number of nodes and there are no edges between each subset of nodes. (colour figure online)

Multilevel networks are networks with a nested hierarchical structure such that nodes may be grouped into subsets of nodes which may further be grouped into subset of subsets of nodes, and so on. It is worth emphasising that the hierarchical nature of a multilevel network corresponds to the grouping of nodes, as opposed to model parameters as might be typical in a Bayesian hierarchical model. A population of networks represents a two-level network such that each subset of nodes correspond to a separate network, with no connections between distinct subsets (see Fig. 1). Wang et al. (2013) proposed ERGMs for multilevel networks, introducing a range of model specifications to account for a range of multilevel structures for two-level networks. Yin and Butts (2022) develop a preprocessing approach to efficiently fit a ’pooled’ ERGM to multiple networks drawn from the same model. Yin et al. (2022) proposed a mixture of ERGMs to model populations of networks in which the group membership is unknown, which was extended to a data-adaptive Dirichlet process mixture of ERGMs by Ren et al. (2023). Schweinberger and Handcock (2015) introduced exponential random graph models with local dependence, providing a general framework encompassing multilevel networks (and thus populations of networks) and establishing a central limit theorem for this class of models.

### ERGMs for brain networks

Exponential random graph models have been applied to resting-state fMRI brain networks (see Simpson et al. (2011) for an early example). Simpson et al. (2012) constructed group-representative networks by taking the mean of the parameter estimates from ERGMs fit to each individual network. Sinke et al. (2016) constructed group-representative networks directly from individual diffusion tensor imaging (DTI) brain networks and then fit Bayesian ERGMs to the resulting group networks. Obando and Fallani (2017) applied ERGMs to functional connectivity brain networks derived from electroencephalographic (EEG) signals. In each of these approaches, the networks are fit independently from each other and, unlike the hierarchical approach described here, there is no pooling of information across networks.

### Other models for populations of networks

Other statistical network models have recently been extended to handle populations of networks. Sweet et al. (2013) proposed a general framework of hierarchical network models (HNMs), which encompasses the model described in this article. They focus on a hierarchical representation of latent space models (Hoff et al. 2002) applied to social networks. Sweet et al. (2014) studied stochastic blockmodel in the HNM framework to infer clusters of nodes shared across networks. Durante et al. (2017) develop an alternative extension of the latent space model (Hoff et al. 2002) to populations of networks based on a low-dimensional mixture model representation. Durante et al. (2018) applied this model in the context of groups of networks to test for differences. Mukherjee et al. (2017) used graphons to detect clusters among multiple networks within a population (as opposed to clusters within networks). Signorelli and Wit (2020) use a model-based clustering method based on generalized linear (mixed) models to cluster networks that share certain network properties of interest.

## Model formulation

### Exponential random graph models

The family of exponential random graph models define probability distributions over the space of networks in terms of sets of summary (or sufficient) statistics. We will focus on the case of undirected, binary networks, with $Y_{ij} = Y_{ji} \in \lbrace 0,1 \rbrace$. Let $\mathcal {Y}$ be the range of $\pmb {Y}$, i.e. the set of all possible outcomes. Let $s(\pmb {y}, \pmb {x})$ denote a vector of *p* summary statistics, such that each component is a function $s_i: \mathcal {Y} \times \mathcal {X}^N \mapsto \mathbb {R}$.

An ERGM is specified by a particular set of *p* summary statistics and a map $\eta : \Theta \mapsto \mathbb {R}^p$. The probability mass function of $\pmb {Y}$ under the corresponding ERGM is given by

1 $$\begin{aligned} \pi (\pmb {y}|, \pmb {x}, \theta ) = \dfrac{\exp \left\{ \eta (\theta )^Ts(\pmb {y}, \pmb {x})\right\} }{Z(\theta )}. \end{aligned}$$

Here, $\theta \in \Theta \subseteq \mathbb {R}^p$ is a vector of *p* model parameters that must be estimated from the data and $Z(\theta ) = \sum _{\pmb {y}' \in \mathcal {Y}} \exp \left\{ \eta (\theta )^Ts(\pmb {y}', \pmb {x})\right\}$ is the normalising constant ensuring the probability mass function sums to one. Given data, that is, a realisation $\pmb {y}$, the goal is to infer which values of $\theta$ best correspond to the data under this distribution. To reduce the notational burden, we will henceforth omit the dependence on the nodal covariates $\pmb {x}$, considering this to be implicit in the specification of the probability distribution.

### A Bayesian multilevel model for populations of networks

The ERGM provides a flexible family of distributions for a *single* network. Our aim is to extend this to a model for a *population* of networks in which each network is accompanied by a set of covariates. To do so, we use ERGMs as the basis of a Bayesian multilevel model. Let $\varvec{Y} = (\pmb {Y}^{(1)}, \dots , \pmb {Y}^{(n)})$ be a set of *n* networks, and let $X \in \mathbb {R}^{n \times q}$ be a matrix of *q* network-level covariates. Identify each network $\pmb {Y}^{(i)}$ with its own vector-valued ERGM parameter $\theta ^{(i)}$. Write $\varvec{\theta } = (\theta ^{(1)}, \dots , \theta ^{(n)})$ for the set of network-level parameters.

We model each individual network $\pmb {Y}^{(i)}$ as an exponential random graph, which we denote $\pmb {Y}^{(i)} \sim \pi (\cdot | \theta ^{(i)})$. Each individual ERGM must consist of the same set of *p* summary statistics $s(\cdot )$. We then propose the following multilevel model:

2 $$\begin{aligned} \begin{aligned} \pmb {Y}^{(i)}&\sim \pi (\cdot | \theta ^{(i)}), ~~ i = 1, \dots , n \\ \theta ^{(i)}&\sim \mathcal {N}\left( x^T_i\beta , \Sigma _\epsilon \right) , ~~ i = 1, \dots , n \end{aligned} \end{aligned}$$

where $\beta$ is a $q \times p$ *matrix* of parameters, and the *q*-vector $x_i$ corresponds to the $i^{th}$ column of the matrix *X*. We assume that, conditional on their respective network-level parameters $\theta ^{(i)}$, the $\pmb {Y}^{(i)}$ are independent. We highlight the connection to multivariate linear regression: we have vector-valued ‘response’ variables $\theta ^{(i)}$ whose dependence on a set of *q* explanatory variables *X* we would like to assess, allowing the components of the residuals $\epsilon ^{(i)}:= \theta ^{(i)}-x^T_i\beta$ to be correlated. The difference with standard multivariate linear regression is that the responses are not observed but are instead latent parameters of an ERGM model. With this specification comes the flexibility associated with multivariate linear regression; the *X* matrix can include polynomial terms and interactions between the covariates of interest.

#### Prior specification

The full conditional likelihood described by (2) can be written

3 $$\begin{aligned} \begin{aligned} p(\varvec{Y} \mid \varvec{\theta }, X, \beta , \Sigma _\epsilon )&= \prod _{i=1}^n p(\pmb {Y}^{(i)} \mid \theta ^{(i)}, x_i, \beta , \Sigma _\epsilon ) \\&= \prod _{i=1}^n \pi (\pmb {Y}^{(i)} \mid \theta ^{(i)})p(\theta ^{(i)} \mid x_i, \beta , \Sigma _\epsilon ). \end{aligned} \end{aligned}$$

To complete this model, we must therefore specify a prior on $(\beta , \Sigma _\epsilon )$. Motivated by computational simplicity, we opt for the (conditional) conjugate prior $p(\beta , \Sigma _\epsilon ) = p(\beta \mid \Sigma _\epsilon )p(\Sigma _\epsilon )$, with

4 $$\begin{aligned} \begin{aligned} \beta \mid \Sigma _\epsilon&\sim \mathcal{M}\mathcal{N}\left( \beta _0, \Lambda _0^{-1}, \Sigma _\epsilon \right) , \\ \Sigma _\epsilon&\sim \mathcal {W}^{-1}\left( V_0, \nu _0 \right) , \end{aligned} \end{aligned}$$

where $\beta _0$ is a $q \times p$ prior mean matrix, $\Lambda _0$ is a $p \times p$ positive definite matrix, $V_0$ is a $q \times q$ positive definite matrix, and $\nu _0 > q - 1$. Here, $\mathcal{M}\mathcal{N}(M, U, V)$ denotes a matrix-normal distribution with location matrix *M*, row-based scale matrix *U*, and column-based scale *V*. $\mathcal {W}^{-1}(\Psi , \nu )$ is an inverse-Wishart distribution with scale $\Psi$ and $\nu$ degrees of freedom.

Equipped with this prior, we can factorise the posterior of $(\beta , \Sigma _\epsilon )$ given the matrix *X* and the network-level parameters $\varvec{\theta }$, into $p(\beta , \Sigma _\epsilon \mid \varvec{\theta }, X) = p(\beta \mid \Sigma _\epsilon , \varvec{\theta }, X)p(\Sigma _\epsilon \mid \varvec{\theta }, X)$ with

5 $$\begin{aligned} \begin{aligned} \beta \mid \Sigma _\epsilon , \varvec{\theta }, X&\sim \mathcal{M}\mathcal{N}\left( \beta _n, \Lambda _n^{-1}, \Sigma _\epsilon \right) , \\ \Sigma _\epsilon \mid \varvec{\theta }, X&\sim \mathcal {W}^{-1}\left( V_n, \nu _n \right) , \end{aligned} \end{aligned}$$

where

6 $$\begin{aligned} \begin{aligned} \nu _n&= \nu _0 + n \\ \Lambda _n&= X^TX + \Lambda _0 \\ \beta _n&= \Lambda _n^{-1}\left( X^T\varvec{\theta } + \Lambda _0\beta _0\right) \\ V_n&= V_0 + \left( \varvec{\theta } - X\beta _n\right) ^T\left( \varvec{\theta } - X\beta _n\right) \\&\quad + \left( \beta _n - \beta _0\right) ^T \Lambda _0 \left( \beta _n - \beta _0\right) . \end{aligned} \end{aligned}$$

Note that conditional on *X* and $\varvec{\theta }$, the networks *Y* are independent of $(\beta , \Sigma _\epsilon )$ and hence do not appear directly in the (conditional) posterior. However, as we shall see below, the networks are present in the posterior for $\varvec{\theta }$. This motivates a Gibbs sampling approach, whereby we iteratively draw from the required conditional distributions.

Regarding the choice of values for the prior hyperparameters $(\beta _0, \Lambda _0, V_0, \nu _0)$, studies of single-network Bayesian ERGMs typically assume relatively flat multivariate normal prior distributions on the model parameters (Caimo and Friel 2011; Sinke et al. 2016; Thiemichen et al. 2016). In this spirit, we suggest default priors of $\beta _0 = 0$ (i.e. the $q \times p$ zero matrix), $\Lambda _0^{-1} = 100I_p$, $V_0 = I_q$, and $n_0 = q + 1$. Informative priors can be used given information from previous studies (see e.g. Caimo et al. (2022), Caimo et al. (2017)) though we note that the appropriate setting of informative priors can be a challenging task due to the typically high levels of dependence between parameters (Koskinen et al. 2013).

## Posterior computation

The double-intractability of the ERGM posterior distribution means that standard MCMC schemes such as the Metropolis algorithm are not suitable. This is due to the presence of the intractable normalising constants $Z(\theta ^{(i)})$ in the denominator, rendering calculation of the Metropolis acceptance rates computationally infeasible. Several methods have been proposed in recent years to perform Bayesian inference in the presence of intractable normalising constants (see Park and Haran (2018) for a review). We focus here on the exchange algorithm (Murray et al. 2006), employed in the context of single-network Bayesian ERGMs by Caimo and Friel (2011). We first recap the exchange algorithm in the context of Bayesian ERGMs before describing a *exchange-within-Gibbs* scheme to generate samples from the joint posterior.

Consider a Metropolis update for a single-network Bayesian ERGM. The acceptance probability for a proposal $\theta '$ from current value $\theta$ requires evaluation of the ratio $Z(\theta ) / Z(\theta ')$, which is computationally intractable. The exchange algorithm is an MCMC scheme designed to circumvent this obstacle. This is achieved by introducing an auxiliary variable $\pmb {y}' \sim \pi (\cdot |\theta ')$, i.e. a network drawn from the same exponential random graph model with parameter $\theta '$.

The algorithm targets an augmented posterior

7 $$\begin{aligned} \pi (\theta , \theta ', \pmb {y}'|\pmb {y}) \propto \pi (\theta |\pmb {y})h(\theta '|\theta )\pi (\pmb {y}'|\theta ') \end{aligned}$$

where $\pi (\theta |\pmb {y})$ is the original (target) posterior, $h(\theta '|\theta )$ is an arbitrary, normalisable proposal function, and $\pi (\pmb {y}'|\theta )$ is the likelihood of the auxiliary variable. For simplicity, we assume $h(\theta '|\theta )$ to be symmetric. Each of the three terms on the right-hand side of Eq. (7) can be normalised, so the left-hand side is well-defined as a probability distribution.

The algorithm proceeds as follows. At each iteration, first perform a Gibbs’ update of $(\theta ', \pmb {y}')$ by drawing $\theta ' \sim h(\cdot | \theta )$ followed by $\pmb {y}' \sim \pi (\cdot |\theta ')$. Next, *exchange* $\theta$ and $\theta '$ with probability $\min (1, AR(\theta ', \theta , \pmb {y}, \pmb {y}'))$, where

8 $$\begin{aligned} \begin{aligned}&AR(\theta ', \theta , \pmb {y}, \pmb {y}') \\&\quad = \dfrac{\pi (\theta '|\pmb {y})}{\pi (\theta |\pmb {y})} \cdot \dfrac{\pi (\pmb {y}'|\theta )}{\pi (\pmb {y}'|\theta ')} \\&\quad = \dfrac{\exp \left\{ \theta '^Ts(\pmb {y})\right\} \pi (\theta ')}{\exp \left\{ \theta ^Ts(\pmb {y})\right\} \pi (\theta )}\dfrac{Z(\theta )}{Z(\theta ')} \cdot \dfrac{\exp \left\{ \theta ^Ts(\pmb {y}')\right\} }{\exp \left\{ \theta '^Ts(\pmb {y}')\right\} }\dfrac{Z(\theta ')}{Z(\theta )} \\&\quad = \exp \left\{ [\theta ' - \theta ]^T[s(\pmb {y}) - s(\pmb {y}')]\right\} \frac{\pi (\theta ')}{\pi (\theta )} \end{aligned} \end{aligned}$$

Crucially, the ratio of intractable normalising constants cancel out, and so this acceptance ratio can indeed be evaluated. The stationary distribution of the Markov chain constructed through this scheme is $\pi (\theta , \theta ', \pmb {y}'|\pmb {y})$ (Murray et al. 2006). Thus, by marginalising out $\theta '$ and $\pmb {y}'$, the algorithm yields samples from the desired posterior, namely $\pi (\theta |\pmb {y})$.

**Algorithm 1:**
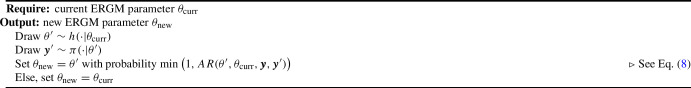
The exchange algorithm update for a Bayesian ERGM (Caimo and Friel 2011)

The exchange algorithm update requires a sample $\pmb {y}'$ from the ERGM $\pi (\cdot |\theta ')$ in order to compute the acceptance ratio. Although perfect sampling for ERGMs is possible, it is computationally impractical except for a few special cases (Butts 2018). A pragmatic alternative, employed in Caimo and Friel (2011) and Wang and Atchadé (2014), is to use the final iteration of a Metropolis–Hastings algorithm as an approximate sample from $\pi (\cdot |\theta ')$ (Hastings 1970; Hunter et al. 2008). A theoretical justification of this approach is given by Everitt (2012): under certain conditions, despite using an approximate sample, the algorithm nevertheless targets an approximation to the correct posterior distribution. Further, this approximation improves as the number of iterations of the inner MCMC increases.

### The exchange-within-Gibbs algorithm

We now extend the exchange algorithm in order to generate samples from our full posterior on a population of networks. As the name suggests, the exchange-within-Gibbs algorithm combines the exchange algorithm with the Gibbs sampler (Geman and Geman 1984) to produce samples from the desired posterior. Note that we can treat the unknown parameters of the model $(\beta , \epsilon , \Sigma _\epsilon )$ as components of a single multi-dimensional parameter. We iteratively sample each component from its conditional distribution given the remaining components.

The full exchange-within-Gibbs scheme is outlined in Algorithm 2. Since each step samples from the respective full conditional distribution, the algorithm ensures that the stationary distribution of the resulting Markov chain is indeed the joint posterior $\pi (\beta , \epsilon , \Sigma _\epsilon |\varvec{y})$ (Tierney 1994). As with the exchange algorithm for the single-network Bayesian ERGM, the most computationally expensive step is sampling $\pmb {y}'$ from $\pi (\cdot |\theta ')$, i.e. simulating an exponential random graph with parameter $\theta ' = X\beta ' + \epsilon '$. Moreover, this step must be performed for each of the individual-level parameter $\theta ^{(i)}$ updates. Thus, the computational cost of each iteration increases linearly with the number of networks in the data. However, these updates may be performed in parallel so, with access to a sufficient number of computing cores, the actual computational time per iteration typically increases sub-linearly with the number of networks.

**Algorithm 2:**
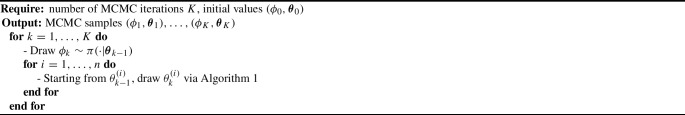
The exchange-within-Gibbs algorithm for a multilevel Bayesian ERGM

#### Choice of parametrisation: centering vs. non-centering

The parametrisation of general multilevel models in the context of MCMC computation has been studied in some detail (Gelfand et al. 1995; Papaspiliopoulos et al. 2003, 2007; Yu and Meng 2011). Here, we discuss the two most commonly used parametrisations: the ‘centered’ and the ‘non-centered’. Let $\mu ^{(i)} = x^T_i\beta$ be the mean for the $i^{th}$ ERGM parameter $\theta ^{(i)}$. The parametrisation presented thus far is known as the centered parametrisation (CP) Gelfand et al. (1995), Papaspiliopoulos et al. (2007), in which the parameters ($\beta , \pmb {\Sigma }_\theta$) are independent of the data $\varvec{Y}$:

9 $$\begin{aligned} \begin{aligned} \pmb {Y}^{(i)}&\sim \pi (\cdot \mid \theta ^{(i)}), ~~~ i = 1, \dots , n \\ \theta ^{(i)}&\sim \mathcal {N}(\mu ^{(i)}, \pmb {\Sigma }_\epsilon ), ~~~ i = 1, \dots , n. \end{aligned} \end{aligned}$$

In contrast, the *non-centred* parametrisation (NCP) can be written as follows:

10 $$\begin{aligned} \begin{aligned} \pmb {Y}^{(i)}&\sim \pi (\cdot \mid \mu ^{(i)} + \epsilon ^{(i)}), ~~~ i = 1, \dots , n \\ \epsilon ^{(i)}&\sim \mathcal {N}(0, \pmb {\Sigma }_\epsilon ), ~~~ i = 1, \dots , n. \end{aligned} \end{aligned}$$

The identity $\theta ^{(i)} = \mu ^{(i)} + \epsilon ^{(i)}$ confirms the equivalence of the two parametrisations. Note that the parameter $\beta$ enters the likelihood directly in (10) via the $\mu ^{(i)}$, so the conditional distribution of $\beta$ given the remaining parameters has an intractable normalising constant $\prod _{i=1}^n Z(x^T_i\beta )$. As above, this can be dealt with via an exchange update, in this case requiring simulation of *n* networks for the normalising constants to cancel in the acceptance ratio, now given by

11 $$\begin{aligned} \begin{aligned}&AR(\beta ', \beta , \varvec{Y}, \varvec{Y}'; \pmb {\Sigma }_\epsilon , X) \\&\quad = \exp \left\{ \sum _{i=1}^n [x^T_i(\beta ' - \beta )]^T[s(\pmb {y}_i) - s(\pmb {y}_i')]\right\} \frac{\pi (\beta ' \mid \pmb {\Sigma }_\epsilon )}{\pi (\beta \mid \pmb {\Sigma }_\epsilon )}. \end{aligned} \nonumber \\ \end{aligned}$$

The centred parametrisation and the non-centred parametrisation tend to be complementary: when one performs poorly, the other tends to perform better (Papaspiliopoulos et al. 2007). More precisely, the centred parametrisation tends to lead to more efficient MCMC performance when $\theta$ is well-identified by the data $\pmb {Y}$, whereas the non-centered parametrisation can be more competitive when $\theta$ is weakly-identified (relative to $(\beta , \pmb {\Sigma }_\epsilon )$). However, if we are in an intermediate setting, and when the parameters of interest are the higher-level parameters $(\beta , \pmb {\Sigma }_\epsilon )$, it is possible to combine both approaches using an ancillarity-sufficiency interweaving strategy (ASIS; Yu and Meng (2011)). ASIS works by combining the updating schemes of the CP and NCP approaches, introducing an intermediate step to first draw ($\beta , \theta$) under the centred parametrisation, and then redrawing the parameters under the non-centered parametrisation. The ASIS algorithm for a multilevel Bayesian ERGM is described in Algorithm 3.

**Algorithm 3:**
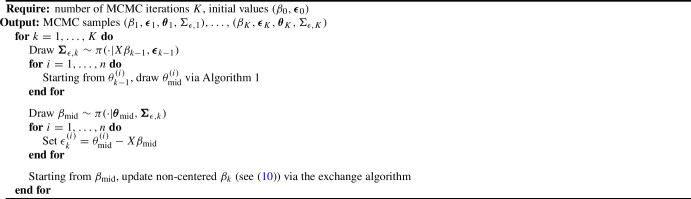
The ASIS algorithm for a multilevel Bayesian ERGM

#### Proposal adaptation

We use multivariate normal random walk proposals in the respective exchange updates of both $\theta ^{(i)}$ and $\beta$, for example

12 $$\begin{aligned} h(\theta ' | \theta _{k-1}) = \mathcal {N}(\theta _{k-1}, \Sigma ) \end{aligned}$$

The choice of the the proposal covariance matrix $\Sigma$ is crucial to the overall efficiency of the MCMC algorithm; we wish to make large proposals that are likely to be accepted in order to explore the posterior in as few iterations as possible. A common approach to tuning covariance proposals for a wide range of random walk based algorithms, including Metropolis-within-Gibbs, is to target an acceptance rate close to 0.234, with acceptance rates between 0.1 and 0.5 often yielding satisfactory results (Roberts et al. 1997, 2001; Roberts and Rosenthal 2009). Since manual tuning of the $n + 1$ proposal covariance matrices would be impractical, we instead implement an adaptive proposal scheme.

For each proposal, we use a version of the adaptive Metropolis algorithm (Haario et al. 2001) considered by Roberts and Rosenthal (2009). Specifically, for the first 1000 iterations, we adapt every 20 iterations, with proposals of the form

13 $$\begin{aligned}{} & {} h_k(\theta ' | \theta _{k-1}) = (1-\gamma ) N \left( \theta _{k-1}, (2.38)^{2} \delta _{k} \Sigma _{k} / p \right) \nonumber \\{} & {} \quad + \gamma N\left( \theta _{k-1}, (0.1)^{2} \delta _{k} I_{p} / p \right) , \end{aligned}$$

where $\Sigma _k$ is the sample covariance matrix of the posterior samples $(\theta _{1},\dots ,\theta _{k-1})$ and $\delta _k$ is an additional scaling factor that is varied to control the magnitude of the proposals. Following Roberts and Rosenthal (2009), we set $\gamma = 0.05$. The role of $\Sigma _k$ is to adapt the direction of the proposals to the MCMC run so far, while $\delta _k$ serves to target an acceptance rate of 0.234. Specifically, we start with $\delta _1 = 1$ and increase (resp. decrease) $\log (\delta _k)$ by $\min (0.5, 1 / \sqrt{(}k)$ if the acceptance rate was below (resp. above) 0.234 in the previous 20 iterations.

### Posterior predictive assessment

Having produced a sufficient number of samples from the posterior distribution, we then assess whether the model adequately describes the data. Since determining the distribution of appropriate test quantities is difficult, assessing such goodness-of-fit for ERGMs is typically performed graphically (Hunter et al. 2008). For a single ERGM fit, one can simulate a large number of networks from the fitted model and compare these ‘posterior predictive networks’ to the observed network. This comparison is usually done via a set of network metrics. If a model fits the data well then the network metrics of the posterior predictive networks should be similar to those of the observed network.

For a population of networks, we can apply the same principles. To do so, we choose uniformly at random *S* values from the posterior samples of $\beta$. For each value, we simulate a network from $\pi (\cdot |X\beta ^{(s)})$. We can then compare these posterior predictive networks to the observed networks based on a set of network metrics. For this purpose, we will use three important network metric distributions that are not explicitly modelled, namely degree distribution, geodesic distance distribution (length of shortest paths) and edge-wise shared partners distribution.

## Results

To illustrate our method, we apply it to a set of simulated networks, demonstrating that it is capable of recovering the ground truth. We also apply our method to resting-state fMRI networks from the Cam-CAN project, a study on healthy ageing (Shafto et al. 2014), to assess how network structure varies with age and fluid intelligence. The R scripts used to generate these results can be found at https://github.com/brieuclehmann/multibergm-scripts.

### Simulation

We generated sets of 30-node networks with nodes split into two ‘hemispheres’ of 15 nodes each. We simulated the networks from an exponential random graph model with three terms: total number of edges (‘edges’), total number of edges between nodes in the same hemisphere (‘nodematch.hemisphere’), and the geometrically-weighted edgewise-shared partner (GWESP) statistic (‘gwesp.fixed.0.9’). The GWESP statistic of a network *y* is a measure of clustering and is given by:

14 $$\begin{aligned} GWESP(\pmb {y}) = e^\tau \sum _{w=1}^N \lbrace 1 - (1 - e^{-\tau })^w \rbrace EP_w(\pmb {y}), \end{aligned}$$

where $EP_w(y)$ is the number of connected node pairs having exactly *w* shared partners and $\tau$ is a decay parameter, which we fix at $\tau = 0.9$. The decay parameter attenuates the effect of the number of higher-order edgewise shared partners relative to lower-order edgewise shared partners.

We simulate networks under three distinct settings, varying the number of networks in each case: (i) a population of networks with no additional covariate information, (ii) a population of networks where each network is associated with a single continuous covariate, and (iii) a population of networks with two subgroups, so that each network is associated with a binary covariate indicating group allocation.

#### No covariate information

To simulate the networks, we first generated individual-level parameters $\theta _i \sim \mathcal {N}(\mu , \Sigma ), ~ i = 1, \dots , n$ where

15 $$\begin{aligned} \mu&= (-3, 0.5, 0.5)^T \end{aligned}$$

16 $$\begin{aligned} \Sigma&= \frac{1}{50}\begin{pmatrix} 1 &{} -0.5 &{} 0 \\ -0.5 &{} 0.5 &{} 0 \\ 0 &{} 0 &{} 0.5 \end{pmatrix}. \end{aligned}$$

We then used the ergm R package (Hunter et al. 2008) to simulate *n* networks $\pmb {y}_i \sim p(\cdot |\theta _i), ~ i = 1, \dots , n$. The simulation procedure is based on an MCMC algorithm, initialised at a network with the prescribed number of nodes and covariates (in this case, hemisphere labels). With these simulated networks, we applied our exchange-within-Gibbs algorithm with ASIS (Algorithm 3) to generate 12,000 posterior samples, adapting the random-walk proposals for the first 1,000 iterations, and discarding the first 2,000 as burn-in.

**Fig. 2:**
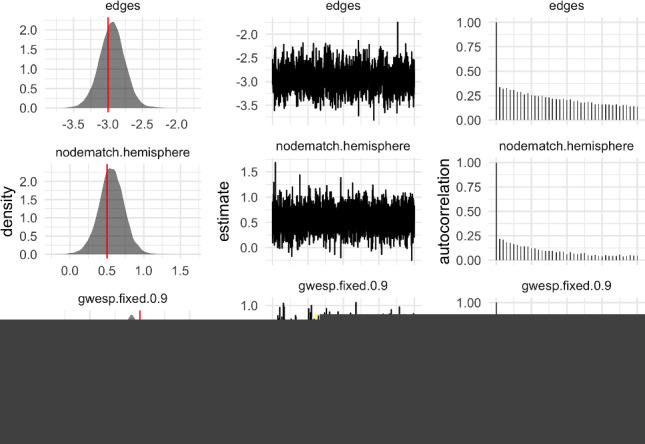
Posterior samples produced by the exchange-within-Gibbs algorithm for the group-level mean parameter, $\mu$, of a single group of ten simulated networks. The true value of $\mu$ is indicated by the red line. (colour figure online)

**Fig. 3:**
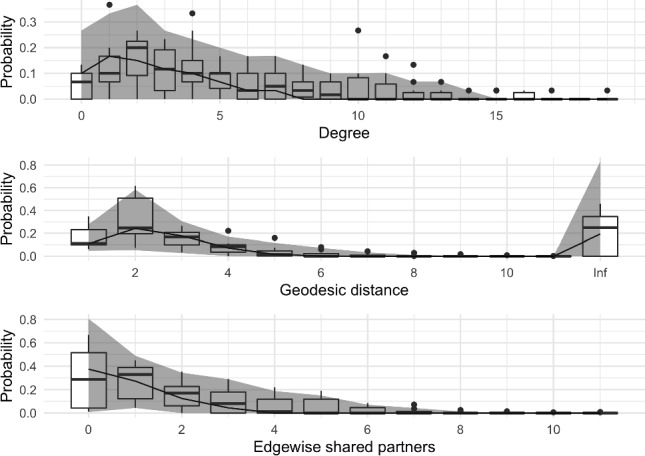
Graphical goodness-of-fit assessment for a single group of ten simulated networks. The box plots correspond to the simulated networks, while the ribbons represent 90% credible intervals corresponding to the posterior predictive networks. Note that a geodesic distance of infinity between two nodes means that there is no path connecting the nodes. (colour figure online)

Figure 2 displays summaries of the posterior samples for the group-level mean parameter $\mu$ of the model fit to $n=10$ networks. The true value of $\mu$ is covered by the posterior density, while the trace and autocorrelation plots indicate that the MCMC has mixed well. To assess the goodness-of-fit, we generated $S=100$ networks from the model at posterior samples of $\mu$ chosen uniformly at random. Figure 3 shows the degree distribution, geodesic distance distribution and edgewise shared partner distribution of these simulated networks against those to which the model was fit.

**Fig. 4:**
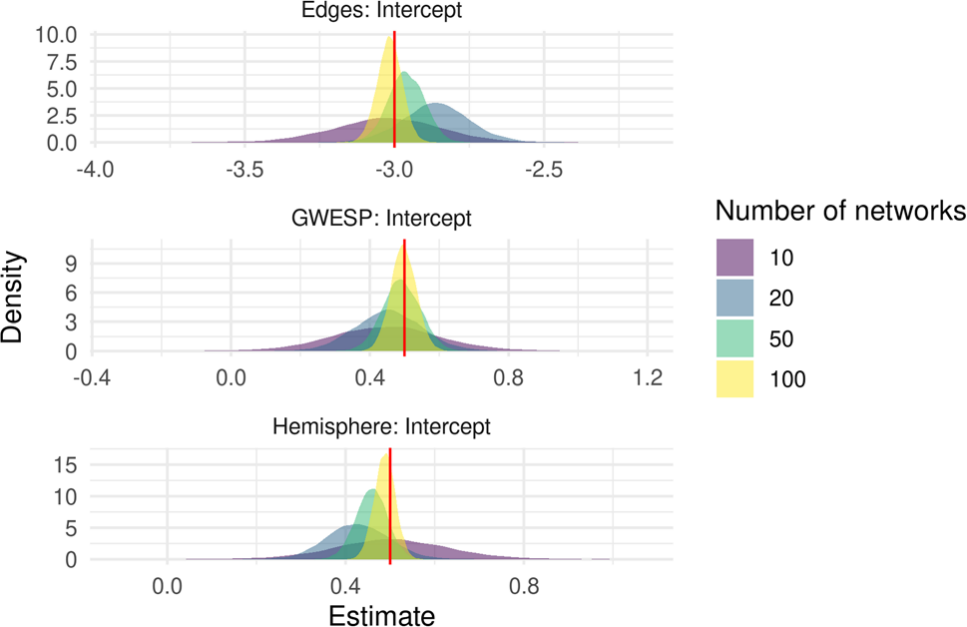
Posterior density plots for the effect parameters $\beta$ in a single group of networks with no covariate information. As the number of networks increases, the posterior concentrates around the true value, depicted by the red vertical line. (colour figure online)

To complete our analysis of a single group of networks, we compare the density of the posterior samples between groups of size $n = 10, 20, 50, 100$. Figure 4 illustrates how the posterior samples of $\beta$ concentrates around the true value as the number of networks in the group increases. We also investigated different settings for the prior hyperparameters ($\Lambda _0^{-1} = 1, 10, 100$ and $\nu _0 = 5, 10, 50$) with $n=10$, finding that these did not have an appreciable effect on the posterior density (Supplementary Figure 2).

As a supplementary analysis, we investigated our model’s performance for increasing network size $N = 30, 60, 90, 120, 150$, where *N* corresponds to the number of nodes in each network. We kept the number of auxiliary MCMC iterations used to simulate each network within the exchange algorithm fixed at $n_{aux} = 1000$. This ensured that the computation time for each of these settings was of similar order, ranging from 50 min for $N=30$ to 90 min for $N=150$ using using 10 Intel(R) Xeon(R) Gold 6140 CPU @ 2.30GHz processors on a computing cluster. However, the number of auxiliary iterations necessary for convergence increases with network size (Krivitsky and Handcock 2014) and hence these auxiliary draws may not adequately represent draws from the desired ERGM required in the exchange algorithm. Supplementary Figure 1 illustrates this, with model performance degrading significantly for $N > 60$.

#### Continuous covariate

We now consider a simulation setting where each network is a associated with a single continuous covariate, such as age. We again consider three cases with $n = 10, 20, 50$ networks in the population, with model matrix $x^T_i = (1, (i-1) / n)$ and $\beta = (a^T, b^T)^T$ where $a = (-3, 0.5, 0.5)^T$ and $b = (-2.6, 0.5, 0.2)^T$, so that the network-level parameter means are uniformly spaced between the vectors *a* and *b*. We then generate $\theta _i \sim \mathcal {N}(\mu _i, \Sigma ), ~ i = 1, \dots , n$ with $\Sigma$ as above, and

$$\begin{aligned} \mu _i = b + \frac{(i - 1)}{n - 1}(a - b), ~~ i = 1, \dots , n. \end{aligned}$$

**Fig. 5:**
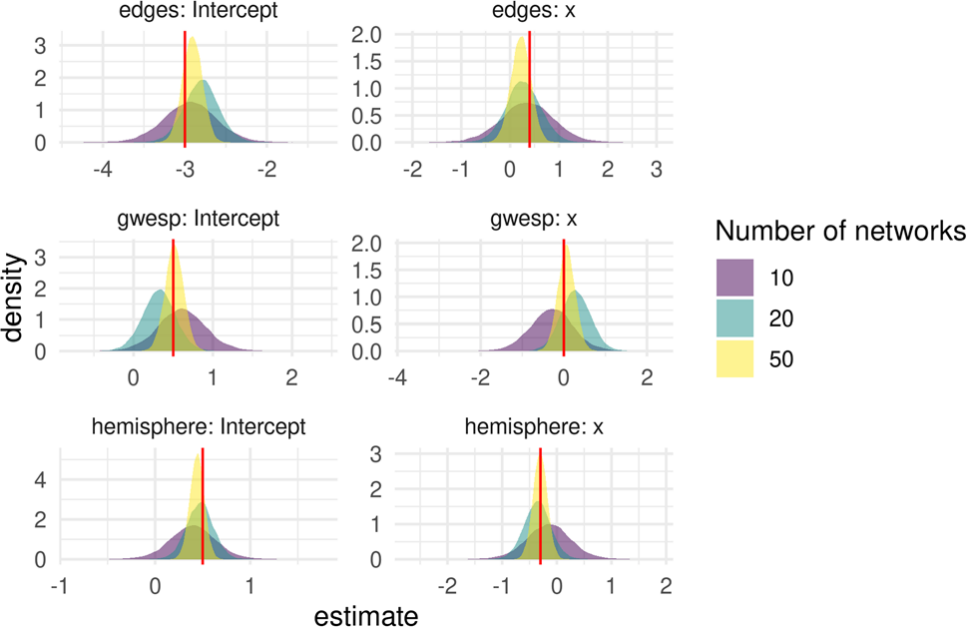
Posterior density plots for the effect parameters $\beta$ in a single group of networks associated with a single continuous covariate *x*. As the number of networks increases, the posterior concentrates around the true value, depicted by the red vertical line. (colour figure online)

Again, the posterior samples of $\beta$ concentrate around the true values for both the intercept and the covariate effect parameters as the number of networks in the group increases (Fig. 5).

#### Binary covariate

To complete our simulation study, we consider a multilevel setting in which the networks are split into two distinct groups $\mathcal {J}_1, \mathcal {J}_2$, so that $x^T_i = (1,0)$ if $i \in \mathcal {J}_1$ and $x^T_i = (0, 1)$ if $\mathcal {J}_2$. We set $\beta = (a^T, (b - a)^T)^T$ so that $\mu _i = a$ if $i \in \mathcal {J}_1$ and $\mu _i = b$ if $i \in \mathcal {J}_2$. As above, we first generated individual-level parameters $\theta _i \sim \mathcal {N}(\mu ^{(g_i)}, \Sigma ), ~ i = 1, \dots , n$, where $g_i \in \lbrace 1, 2 \rbrace$ denotes the group membership of the $i^{th}$ network, and then simulated networks $\pmb {y}_i \sim p(\cdot |\theta _i), ~ i = 1, \dots , n$. We considered a range of numbers of networks, $n = 10, 20, 50$ each of the two groups. The true values were

17 $$\begin{aligned} \mu ^{(1)}&= (-3, 0.5, 0.5)^T \end{aligned}$$

18 $$\begin{aligned} \mu ^{(2)}&= (-2.6, 0.5, 0.2)^T \end{aligned}$$

19 $$\begin{aligned} \Sigma&= \frac{1}{50}\begin{pmatrix} 1 &{} -0.5 &{} 0 \\ -0.5 &{} 0.5 &{} 0 \\ 0 &{} 0 &{} 0.5 \end{pmatrix}. \end{aligned}$$

**Fig. 6:**
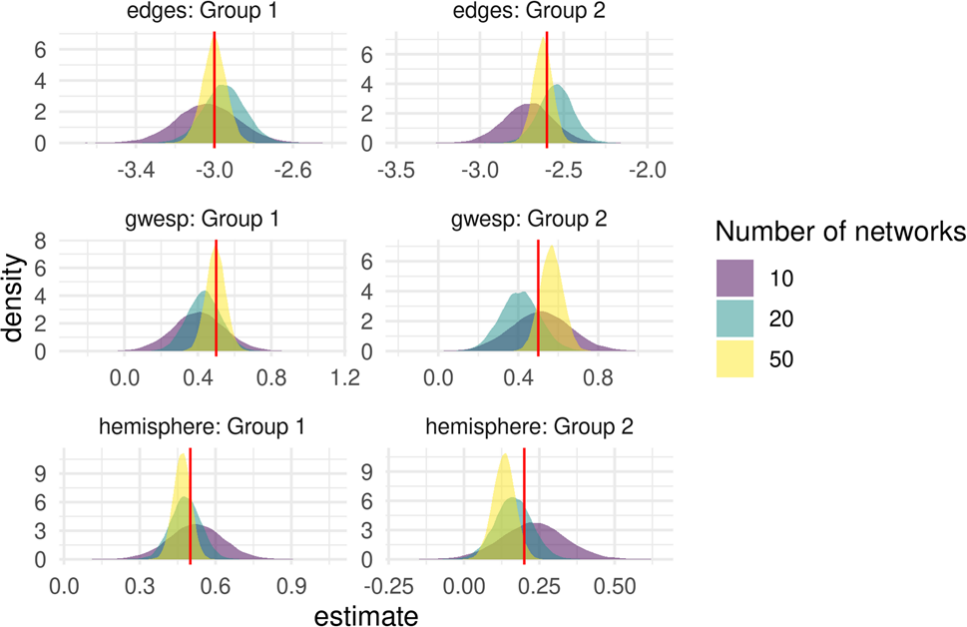
Posterior density plots for a two-group model with $n = 10,20,50$ simulated networks in each group. (colour figure online)

Figure 6 shows the density of the posterior samples for the group-level parameters $(\mu ^{(1)}, \mu ^{(2)})$ for increasing number of networks *n* per group. We see that, as in the single-group setting, the posteriors concentrate around the true values for each group as the number of networks increases.

### Application to human functional connectivity brain networks

We now turn our attention to a real data example: networks derived from resting-state fMRI scans of human brains from the Cambridge Centre for Ageing and Neuroscience (Cam-CAN) research project Shafto et al. (2014), a study on the effect of healthy ageing on cognitive and brain function. The Cam-CAN dataset consists of a range of cognitive tests and functional neuroimaging experiments for approximately 650 healthy individuals aged 18–87. Our aim will be to assess how the functional connectivity structure of the brain varies with age and fluid intelligence, as measured by the Cattell score.

Full details of data collection and preprocessing can be found in Lehmann et al. (2021). To summarise, both structural (T1 and T2) and eyes-closed, resting-state fMRI scans (261 volumes, lasting 8min 40 s) were acquired for each individual. The fMRI scans were motion-corrected and co-registered to the respective structural scans and then mapped to the common Montreal Neurological Institute (MNI) template to ensure comparability across individuals. The fMRI time series were then extracted from 90 cortical and subcortical regions of interest (ROIs) from the AAL atlas (Tzourio-Mazoyer et al. 2002) and adjusted for various confounds using the optimised pipeline of Geerligs et al. (2017).

To construct networks for each individual, we followed a thresholded correlation matrix approach. For individual *i*, we computed the pairwise Pearson correlation between each of the $N = 90$ preprocessed time series, yielding a $N \times N$ correlation matrix $\pmb {C}^{(i)}$. We then applied a threshold *r* to $\pmb {C}^{(i)}$ to produce an $N \times N$ adjacency matrix, $\pmb {A}^{(i)}$, with entries:

20 $$\begin{aligned} \pmb {A}^{(i)}_{kl} = {\left\{ \begin{array}{ll} 1 &{}\text {if } \pmb {C}^{(i)}_{kl} \ge r \quad k,l=1,\dots ,N\\ 0 &{}\text {otherwise.} \end{array}\right. } \end{aligned}$$

The adjacency matrix defines an individual’s network, $\pmb {y}^{(i)}$, with an edge between nodes *k* and *l* if and only if $\pmb {A}^{(i)}_{kl}=1$. The threshold *r* was chosen to yield an average node degree of 3 across all the networks, as recommended by Fallani et al. (2017). See Table 1 for summary statistics on the resulting networks for these individuals, as well as their ages and Cattell scores.

**Table 1:** Summary of age, Cattell score, network density, and network transitivity for all individuals in the Cam-CAN fMRI dataset, as well as the youngest 100 individuals, and the oldest 100 individuals

			All	Young	Old
	Age	Mean	53.53	27.45	24.98
		SD	18.23	4.19	5.83
		Min	18.47	18.47	12.00
		Max	88.90	33.45	38.00
	Cattell score	Mean	32.24	37.23	24.98
		SD	6.64	3.64	5.83
		Min	12.00	26.00	12.00
		Max	44.00	44.00	38.00
	Network density	Mean	0.03	0.04	0.03
		SD	0.02	0.02	0.01
		Min	0.01	0.01	0.01
		Max	0.13	0.13	0.08
	Network transitivity	Mean	0.56	0.56	0.55
		SD	0.10	0.09	0.09
		Min	0.00	0.28	0.18
		Max	0.88	0.88	0.82

Cattell scores were missing for 14 out of the 587 individuals

We model the population of networks using the framework described in Sect. 2.2 with an exponential random graph model with four terms: total number of edges (‘edges’), total number of edges between nodes in the same hemisphere (‘nodematch.hemisphere’), total number of edges between homotopic nodes (mirror ROIs in each hemisphere; ‘nodematch.homotopy’) and the geometrically-weighted edgewise-shared partner (GWESP) statistic with decay parameter $\tau = 0.9$ (‘gwesp.fixed.0.9’).

#### Young vs. old

We first turn our attention to an age-only analysis, comparing the functional connectivity network structure between the 100 youngest individuals, indexed $\mathcal {J}_{\text {young}}$, aged 18-33, and the 100 oldest individuals, $\mathcal {J}_{\text {old}}$, aged 74–87. As in the simulation experiment with a binary covariate, we have $x^T_i = (1,0)$ if $i \in \mathcal {J}_{\text {young}}$ and $x^T_i = (0, 1)$ if $\mathcal {J}_{\text {old}}$.

**Fig. 7:**
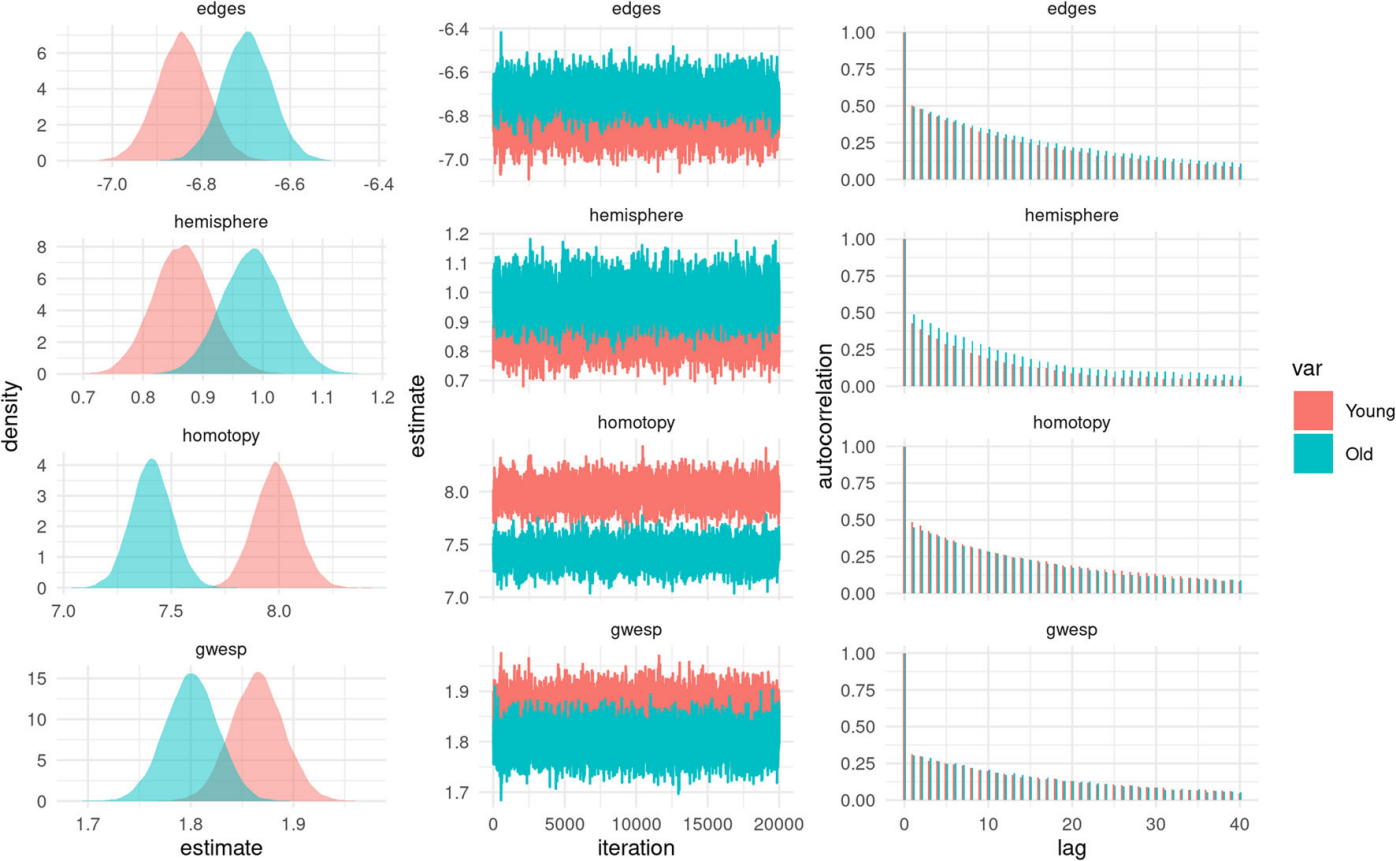
MCMC output from the exchange-within-Gibbs algorithm for the group-level mean parameters of a population of resting-state fMRI networks from a group of 100 young individuals and a group of 100 old individuals. (colour figure online)

We used the exchange-within-Gibbs algorithm with ASIS to generate 22,000 posterior samples, discarding the first 2,000 samples as burn-in. Figure 7 shows summaries of the posterior samples for $(\mu ^{(1)}, \mu ^{(2)})$, with the trace and autocorrelation plots demonstrating that the MCMC has mixed well. The posterior density plots show that the clearest difference between the old group and the young group was the difference in the parameter associated with the number of edges between homotopic nodes (‘nodematch.homotopy’). While this parameter is large and positive for both groups, it is moderately smaller in the old group, indicating that the propensity for homotopic connections is lower in old age. On the other hand, there is no clear evidence for group differences in the remaining parameters. The edges parameters are large and negative, pointing to the overall sparsity of the networks; the intrahemisphere parameters (‘nodematch.hemisphere’) are small and positive, indicating a moderate propensity for connections between nodes in the same half of the brain; and the GWESP parameters are also positive, indicating a propensity to form triangles and thus a degree of functional segregation (Bullmore and Sporns 2009).

**Fig. 8:**
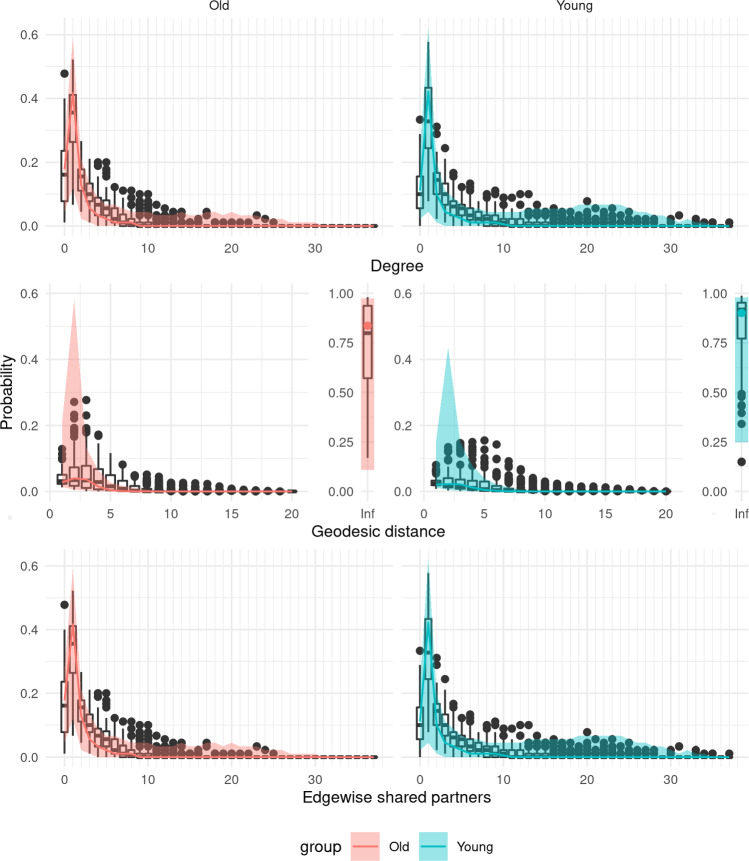
Graphical goodness-of-fit assessment for resting-state fMRI networks from a young group and an old group, fitted in a joint model. The box plots correspond to the observed networks, while the ribbons represent 95% credible intervals corresponding to the posterior predictive networks. Note that a geodesic distance of infinity between two nodes means that there is no path connecting the nodes. (colour figure online)

To assess goodness-of-fit, for both groups we generated $S=100$ networks from the model at posterior samples of $\mu ^{(j)}$ chosen uniformly at random. Figure 8 indicates a reasonable fit for both groups, with the geodesic distance and edgewise shared partner distributions showing a good correspondence between the simulated networks and the observed networks. There appears to be a slight discrepancy in the degree distributions, with the simulated networks in the young group in particular having fewer nodes of degree 4 to 6 relative to the observed networks.

#### Age and fluid intelligence

Finally, we consider a model that jointly assesses the effect of age and fluid intelligence on the brain’s functional connectivity structure. We use the same ERGM summary statistics as before - edges, ‘nodematch.hemisphere’, ‘nodematch.homotopy’, GWESP - and set

$$\begin{aligned} x^T_i = (1, \text {age}_i, \text {IQ}_i, \text {age}_i*\text {IQ}_i), \end{aligned}$$

where $\text {age}_i$ and $\text {IQ}_i$ denote the age and Cattell score (a measure of fluid intelligence), respectively, of individual *i*. For this model, we took a subset of 100 individuals across the range of non-missing Cattell scores. We highlight the use of the interaction term between age and fluid intelligence to capture the joint effect of these two covariates over and above their corresponding main effects. We again used the exchange-within-Gibbs algorithm with ASIS to generate 22,000 posterior samples, and discarded the first 2,000 samples as burn-in.

**Fig. 9:**
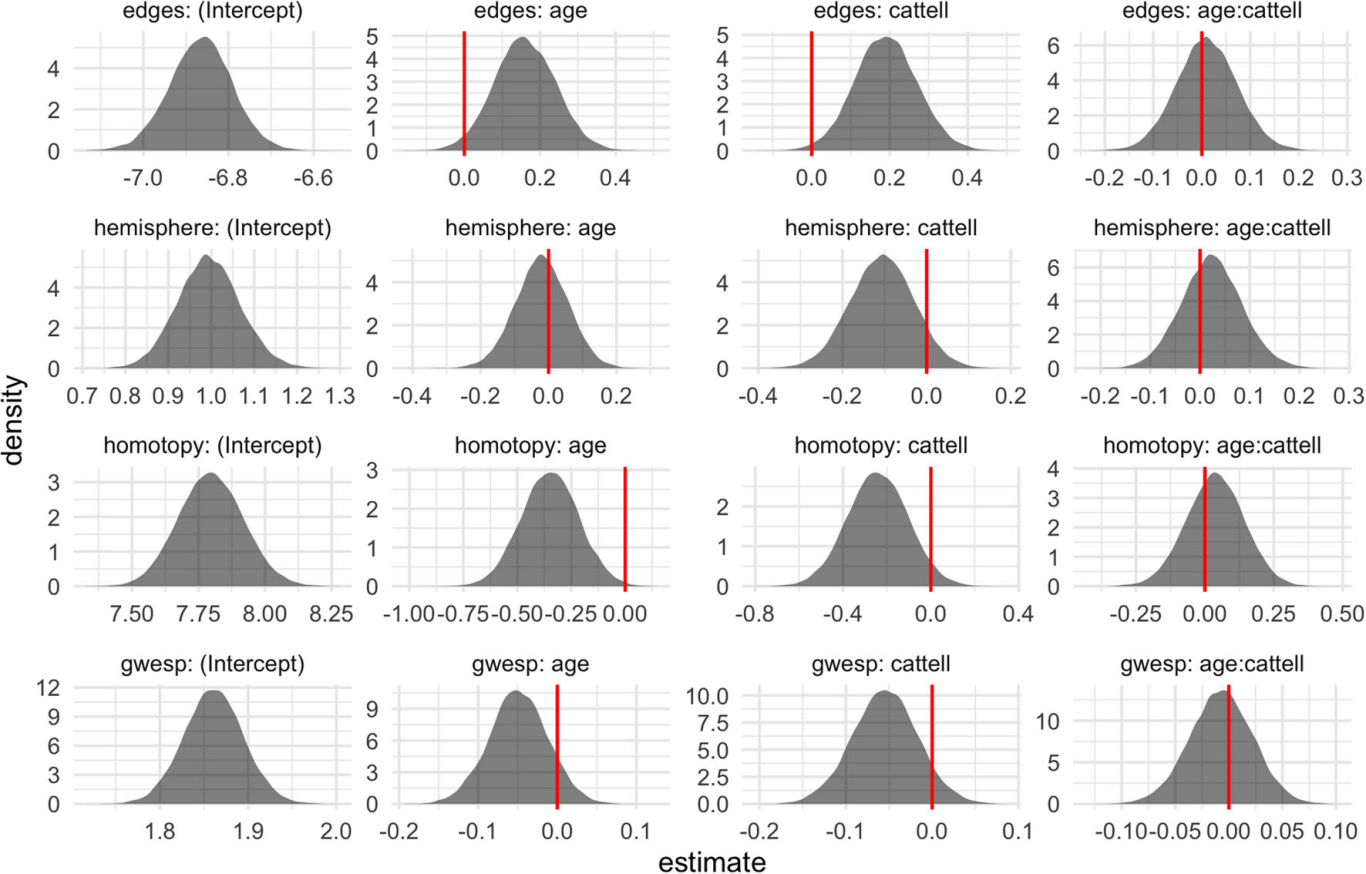
MCMC output from the exchange-within-Gibbs algorithm for the effect parameters of a population of 100 resting-state fMRI networks. (colour figure online)

Figure 9 shows the density plots for the resulting posterior samples. As with the previous analysis comparing a group of young individuals and a group of old individuals, the clearest age-related effect was associated with the number of edges between homotopic nodes. Higher fluid intelligence, as measured by the Cattell score, was associated with a higher propensity for the total number of edges, but a lower propensity for both intrahemispheric connections and homotopic connections. Reduced homotopic connectivity has previously been observed in rs-fMRI networks, with evidence suggesting that reduced synchrony between brain hemispheres at rest may be predictive of higher intelligence (Santarnecchi et al. 2015). The parameter estimates for the age - fluid intelligence interaction term were centred around zero, indicating no additional effect on top of the additive effects associated with age and fluid intelligence separately. To explore possible non-linear effects of age and fluid intelligence, we also fit a model with quadratic terms for age and Cattell score, finding no quadratic effects for age but a small quadratic effects on intrahemispheric connections (positive) and triangle propensity (GWESP; negative) (Supplementary Figure 3).

## Discussion

The main contribution of this article is to introduce a multilevel framework for modelling populations of networks with network-level covariate information, along with a novel MCMC procedure for performing inference with the framework. While the framework itself is a natural multilevel extension of a single ERGMs, the inference procedure is more involved due to the intractability of the ERGMs likelihood and the challenges associated with MCMC for hierarchical models. We have presented how our framework can be applied to resting-state fMRI data to assess how the brain’s functional connectivity network structure varies with age and intelligence score. Although we chose here to focus on networks constructed from resting-state fMRI scans, our framework could also be applied to networks derived from other neuroimaging modalities such as magnetoencephalography (MEG) or diffusion tensor imaging (DTI).

An important extension to the framework would be to use weighted exponential random graph models (Krivitsky 2012; Desmarais and Cranmer 2012). These are an extension of the binary ERGMs that can be applied to weighted networks, thus avoiding the thresholding step in the construction of functional connectivity networks. Indeed, one version of a weighted ERGMs, the generalised exponential random graph model (GERGM) (Desmarais and Cranmer 2012) was recently applied to a 20-node functional connectivity network (Stillman et al. 2017). This approach has the additional advantage of modelling the mean connectivity directly and thus would avoid any confounding due to differences in mean connectivity. However, the GERGM is at present extremely computational intensive, rendering it infeasible for a population of networks.

One of the key challenges in applying our framework to real data is the choice of which network summary statistics to include in the model. A fully Bayesian model selection method based on reversible-jump MCMC has been developed for exponential random graph models on single networks (Caimo and Friel 2013). A similar approach could be developed for our framework, though the computational cost is likely to be prohibitive. A more pragmatic approach would be to develop a graphical goodness-of-fit method by comparing the posterior predictive distributions under different models. More flexible specifications of the relationship between the covariates and ERGM parameters, such as spline-based models, would also be a fruitful avenue for future work.

The computational cost of our MCMC algorithm is considerable. Even with a 20-core computing cluster (Intel(R) Xeon(R) Gold 6140 CPU @ 2.30GHz), the algorithm took over 5 h to produce the 22,000 posterior samples in the real data example presented above. The main computational bottleneck lies in simulating the exponential random graphs at each MCMC iteration. While the computational cost should increase roughly linearly in the number of networks, Krivitsky and Handcock (2014) provide empirical evidence indicating that the cost may grow on the order of $p(N + E)\log (E)$ where *p* is the number of summary statistics, *N* is the number of nodes, and *E* is the number of edges. It may be possible to reduce the number of ERGMs simulations at each MCMC iteration using noisy Monte Carlo methods (Alquier et al. 2016). Other promising avenues include variational inference for ERGMs (Tan and Friel 2020), or pseudolikelihood methods (Bouranis et al. 2017), which could both be extended to our framework to yield approximate Bayesian inference at a much reduced computational cost relative to MCMC.

### Supplementary Information

Below is the link to the electronic supplementary material.

Supplementary file 1 (pdf 464 KB)
