# Supplementary material for: A Bayesian multilevel model for populations of networks using exponential-family random graphs
Source: Stat Comput. 2024 Jun 19;34(4):136. doi: 10.1007/s11222-024-10446-0 (PMC11186958; doi:10.1007/s11222-024-10446-0)
Supplement: Supplementary file 1 — (pdf 464 KB) [file 11222_2024_10446_MOESM1_ESM.pdf]

Supplementary material for *A Bayesian multilevel model for populations of networks using exponential-family random graphs*

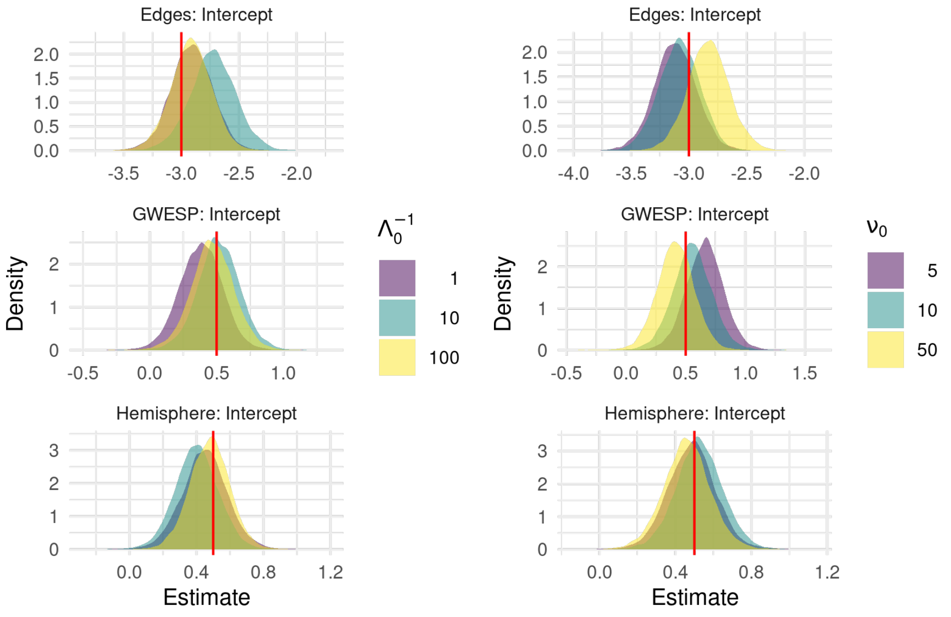

Fig. S1 Posterior density plots for the effect parameters β in a single group of $n = 10$ networks with no covariate information, under different settings for the prior hyperparameters ($\Lambda_0^{-1} = 1, 10, 100$ and $\nu_0 = 5, 10, 50$). To ensure the same prior mean for Σ_ϵ, we set $V_0 = (\nu_0 - p - 1)I_q$, where here the number of summary statistics is $p = 3$ and the number of covariates is $q = 1$.

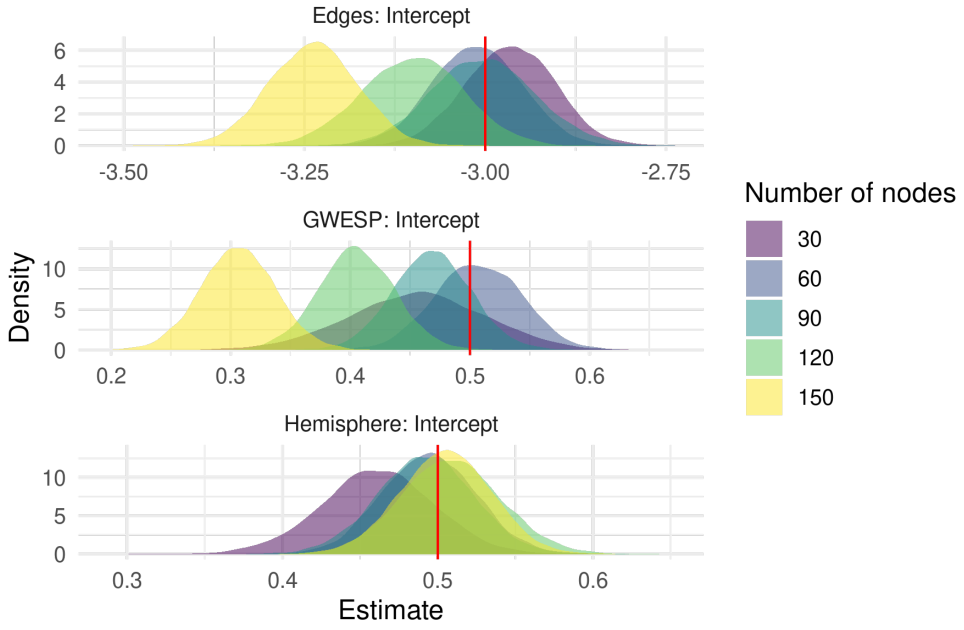

Fig. S2 Posterior density plots for the effect parameters β in a single group of networks with no covariate information. As the number of nodes in the networks increases, the model fit degrades.

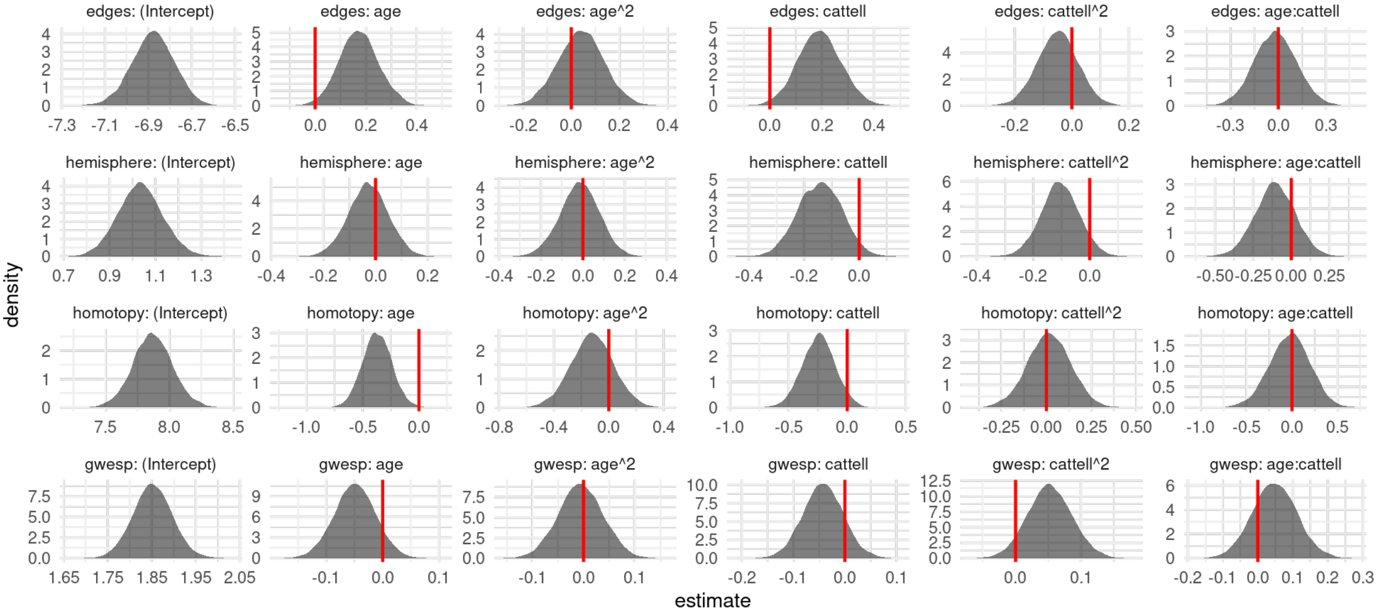

Fig. S3 MCMC output from the exchange-within-Gibbs algorithm for the effect parameters of a population of 100 resting-state fMRI networks, including quadratic covariate terms.
